# Perceived social support and compliance with stay-at-home orders during the COVID-19 outbreak: evidence from Iran

**DOI:** 10.1186/s12889-020-09759-2

**Published:** 2020-11-04

**Authors:** Toktam Paykani, Gregory D. Zimet, Reza Esmaeili, Amir Reza Khajedaluee, Mohammad Khajedaluee

**Affiliations:** 1 Social Development and Health Promotion Research Center, Gonabad University of Medical Sciences, Gonabad, Iran; 2 Department of Pediatrics, Indiana University School of Medicine, Indianapolis, IN USA; 3 Faculty of Medicine, Mashhad University of Medical Sciences, Mashhad, Iran; 4 Department of Community Medicine, Faculty of Medicine, Mashhad University of Medical Sciences, Mashhad, Iran

**Keywords:** Social support, Social distancing, Self-isolation, COVID-19

## Abstract

**Background:**

Strong evidence demonstrates that social support plays a key role in facilitating preventive health behaviors. The aim of the current study was to assess the effects of perceived social support on compliance with stay-at-home orders in response to a COVID-19 outbreak during the Persian New Year (Nowruz) holydays, since Nowruz holidays of 2020 coincided with the peak of the coronavirus epidemic in Iran.

**Methods:**

This cross-sectional survey was carried out based on phone interviews of 1073 adults aged over 18 years from 4 to 12 April 2020 in Mashhad, Khorasan-Razavi Province, as the second largest city of Iran. A systematic random sampling was carried out using fixed phone number lists provided by Telecommunication Company of Khorasan-Razavi Province. Phone interviews were carried out by trained interviewers from the Iranian Students Polling Agency (ISPA) at various times of the day. The survey included sociodemographic questions, perceived social support scale (MSPSS) and questions about self-isolation during the Nowruz holiday. Statistical analysis included Chi-square test, Mann-Whitney test and multivariate logistic regression.

**Results:**

20.5% of participants reported poor compliance with stay at home orders during the first 2 weeks of Nowruz. Clear social gradients were not found in stay-at-home compliance. When controlling socio-demographic factors, perceived social support, interestingly, both fostered and hindered people’s compliance with stay at home orders, depending on the source of support from family members (OR = .874, 95% CI = .803, .950, *p* < .005), friends (OR = 1.147, 95% CI = 1.076, 1.222, *p* < .001) and a significant other person (*OR = .926, 95% CI = .849, 1.010, p = .084*).

**Conclusions:**

Public health messaging may need to emphasize the role that friends and families can play in helping to protect those in their friendship/family groups by promoting compliance with social distancing. Further in-depth studies are recommended to evaluate how this kind of messaging can most effectively encourage people to engage in social distancing practices.

## Introduction

At the end of 2019, an outbreak of novel Coronavirus (COVID-19) occurred in Wuhan, China. The outbreak spread rapidly across the globe and was announced as a pandemic by the World Health Organization (WHO) on March 11, 2020 [1]. In Iran, the first official announcement of deaths due to COVID-19 was published on Feb 19, 2020 [2]. COVID-19 quickly spread throughout the entire country, and as of March 19, 2020, Iran reported 18,407 COVID-19 laboratory-verified cases and 1284 associated deaths. Regional statistics of WHO have shown that the highest COVID-19 laboratory-verified cases and its associated deaths are reported by Iran within WHO-EMRO countries [3]. Iran was estimated to reach its peak number of COVID-19 cases at the beginning of the Persian New Year; Nowruz (20 March). Nowruz (literally new day); in which, Iranians and other Persian-speaking nations celebrate the end of winter. In Iran, the festival routinely lasts 1–2 weeks. Celebrating the festival may exacerbate outbreaks since Nowruz is the time of catching up with families, friends and other people. During Nowruz, people visit close relatives and friends, exchange gifts and feast. The number of daily person-to-person contacts for a typical person may increase up to 20 fold during the national festival. Based on the traditions, the elderly members of the family (grandparents) are visited first. A typical family may be visited by 50–100 relatives during Nowruz. Furthermore, Nowruz is a high travel season [4]. However, the current evidence indicates that the most effective way to control the outbreak is use of social distancing measures to break the chain of infection transmission [2].

Normally, social distancing imposes a large economic pressure on the nation and the government. This economic pressure is worse for the third world countries such as Iran, which has long been under constant pressure of strict sanctions [2, 4]. However, keeping mortality as low as possible is the highest priority. Hence, following the COVID-19 outbreak at the end of winter, the Iranian government asked people to stay-at-home, ordered social distancing and closed schools, universities, libraries and museums. Mass gathering events such as religious gatherings, conferences, cultural celebrations and music festivals were cancelled or postponed. All public places, except pharmacies, bakeries, groceries and gas stations, were ordered to shut down. The working hours in most government offices and banks decreased. Traffic plans were regulated and public transport systems, i.e. subways and bus services were closed in most cities, including Mashhad (from March 15, 2020). Moreover, the government limited travel during Nowruz. Despite such mitigation measures and strong recommendations urging people to stay at home as much as possible, some people did not practice social distancing and left their houses for inessential activities. Hence, COVID-19 quickly spread throughout the entire country despite all national containment efforts. The COVID-19 outbreak rose, with 55,743 laboratory-verified cases and more than 3452 deaths on April 4, 2020 [5].

Little is known regarding the factors affecting compliance with health care advice during pandemics. Considering the growing body of literature highlighting the role of social support for health behavior change [6, 7], the current study aimed to assess associations between perceived social support and the level of compliance with stay-at-home advisories during the 2020 Nowruz holiday among residents of Mashhad, Iran.

### Perceived social support and health protective behaviors

Social support is generally described as the availability of reliable people, who let us know that they care about, value, and love us [8]. Social support includes support perceptions (perceived support) and supportive behaviors (received support), which can promote overall well-being as well as increasing personal resistance to health problems [9]. Perceived social support is the personal subjective appraisal of the availability and adequacy of resources and reactions provided by their social networks. Received social support refers to objective appraisals of personal social connections and their consequent functions [10].

Social support may come from different sources, e.g. family, friends, romantic partners, community ties, and colleague [11]. Social networks affect health behaviors by several mechanisms. Social contacts provide information on resources and products, which can be used to change a usual behavior. Furthermore, social networks provide social capital or how-to information, which can be used to carry out jobs [10]. Literatures are now available, describing roles of perceived social support in affecting positive psychological outcomes such as self-efficacy, self-esteem and resilience. However, these may contribute to promote health behaviors [6]. In recent years, investigations on social support as a factor linked to treatment adherence have increased. Good examples of this increase included investigations on patients with obesity [12], hypertension [10], type-2 diabetes [12] and HIV [13]. However, findings are sometimes controversial [6].

Social support can greatly contribute to physical and mental health. Researchers have found that supportive family environments were linked to various preventive health practices by elderly people. Umberson (1987) showed that support could promote preventive health behaviors via direct and indirect social controls and suggested that health is a normative circumstance and behaviors that contributed to morbidity and mortality are deviant behaviors. Therefore, direct social control might occur via external bans on unconventional or deviant behaviors [14]. A number of studies have been carried out on the association of social support with stress and coping during outbreaks such as influenza [15], Ebola [16], SARS [17], and COVID-19 [18, 19]. However, there is little or no published research on the role of social support in promoting public compliance with social distancing orders as the most effective way of limiting spread of communicable viruses. Therefore, the aim of the current study was to answer the following research questions.

### Research questions

1) Is there a social gradient in participants’ compliance with self-isolation after controlling for demographic characteristics? and 2) Is perceived social support positively linked to compliance with stay at home orders after accounting for participants’ demographic and socioeconomic characteristics?

## Materials and methods

### Data collection

Data were collected in Mashhad, Khorasan-Razavi Province, Northeastern Iran, through phone interviews of 1073 adults aged over 18 years, from 4 to 12 April 2020. The phone survey was carried out using a fixed phone number list provided by the Telecommunication Company of Iran (TCI), Khorasan-Razavi Headquarter. A random systematic sampling was carried out to select participants for phone interviews. A total of 3200 calls were made, of which 1669 failed (busy, no answer, on fax or line block). Unavailable phone numbers after five attempts were removed from the list. A total of 1531 individuals answered the phone calls. Of these individuals, 223 were excluded (aged < 18 years) and 235 refused to participate. Hence, 1073 adults participated in this study. The phone interviews were carried out by trained and experienced data collection staff from the Iranian Students Polling Agency (ISPA), affiliated to the Academic Center for Education, Culture and Research (ACECR). A supervisor monitored data collection. The interviewers were informed that interviews would be monitored, yet they did not know when these observations occurred. They were monitored randomly and more than 60% of the calls by each interviewer were observed. Interviewers made the phone calls at various times of the day. At the beginning of each interview, major objectives of the study were briefly explained to the participant to receive their verbal participation consent. Inclusion criteria were age 18 years or over, being a resident of Mashhad, willingness to participate in the study, and understanding Persian language. The study was approved by the Ethics Committee of Mashhad University of Medical Sciences, Mashhad, Iran.

### Measurements

#### Socioeconomic status

Income, education level and social class (subjective) of the participants were considered as socioeconomic factors. Social surveys in Iranian society include a number of challenges when aiming at a reliable estimate of income or wealth since most people are not willing to share their income information. Thus, asking for income disclosure results in high proportions of missing values [20]. Based on previous studies that subjective measures could be valid indicators [21], household income was assessed using 5-point scale, ranging from “very difficult” to “very easy” that showed the respondents’ feelings about their household economic situations [22]. Another proxy for socioeconomic status was education. In this study, education was assessed using the highest educational degree received by the participants based on the International Standard Classification of Education (ISCED-97). Then, education levels were categorized into three major categories: low level included under secondary level (ISCED 0–2), medium or second stage of secondary level (ISCED 3) and high level or third level (ISCED 5–7). Social class was another socioeconomic indicator. Subjective social class was identified by asking the participants’ perception of their social class relative to other people [23]. This was rated 1 (upper class) to 5 (lower class). Responses of lower class, working class and lower middle class were recoded as low and upper middle class and responses of upper class were recoded as high class.

#### Perceived social support

Several instruments are available to assess social support. A promising scale widely used for decades is the Multidimensional Scale of Perceived Social Support (MSPSS) originally published by Zimet et al. in 1988 [24]. The MSPSS is a 12-item scale that assesses perceived support from three sources of family, friends and a significant other person (e.g., spouse or best friend) using 7-point Likert-scale, ranging from 1 as very strongly disagree to 7 as very strongly agree. The MSPSS assesses both perceived availability and adequacy of emotional and instrumental support. This instrument is brief, easy to administer, and has been found to be reliable and valid in various populations and languages. The reliability and validity of the Persian translation of the MSPSS was demonstrated in a previous study [25].

#### Compliance with stay at home orders

Personal compliance with stay at home orders was assessed using a single screening question [26] linked to the degree to which, the participants were isolating themselves from non-household members: “In the past two weeks (Nowruz holiday), to what extent did you limit your in-person contact with people outside your household?” This item was scored on a 5-point scale, ranging from 1 “not at all” to 5 “a great deal”. The original five-point response scale was dichotomized: “not at all” “a little” and “somewhat” responses were recoded as one and “a lot” and “a great deal” responses were recoded as zero.

Reliability of the single-item self-report of stay-at-home compliance was assessed in a randomly selected sub-sample (*n* = 120). Test-retest reliability using 2-week intervals between the assessments was moderate (κ = 0.56, 95% CI (0.43–0.67)).

Participants were also asked “how many times did you leave home for each of the following purpose during the last week? Going to workplaces, daily shopping of necessities, meeting relatives or friends, going to banks or other institutes, doing exercises and recreations, going to pharmacies or health centers, and others”. Answers were provided based on a 3-point scale with answer options of never or once, two or three times and more than three times.

### Statistical analysis

Descriptive statistics for categorical variables were described using frequencies and percentages. Continuous variables were summarized as means and standard deviations (SDs). Differences in socio-demographic characteristics between the participants who complied or did not comply with social isolation were assessed using Pearson chi-squared test (χ2) for the categorical variables such as sex, marital status, education, occupation and income and Mann-Whitney test for continuous variables such as age and persevered social support. Multivariate logistic regression analysis was used to assess effects of demographic characteristics, socioeconomic factors and social support on compliance with self-isolation. Overall, two models were built according to the research questions. The first model included demographic and socioeconomic factors. Model 2 was built on model 1 by adding perceived social support variables to estimate the effect of perceived social support from different sources on compliance with self-isolation. Odds ratios (ORs) and their confidence intervals are reported. The significance level was set at 0.05. Statistical analysis was carried out using Stata 13.0 (Stata Corporation, Texas, USA).

## Results

Descriptive statistics for all study variables are summarized in Table 1.

**Table 1:** Participant characteristics by compliance with stay at home orders (*n* = 1073)

	Total	good compliance	Poor compliance	(χ^2^ test, df) or z-score, *P*-value
	N (%) or median (IQR)	N (%) or median (IQR)	N (%) or median (IQR)	
Overall sample	1073 (100)	853 (79.5)	220 (20.50)	
Age (year)	38 (30,51)	39 (30,52)	36 (30,48)	1.88 *P* = .059
**Gender**				
Female	547 (50.98)	472 (43.99)	75 (6.99)	(31.58,1) *P* < .001
Male	526 (49.02)	381 (35.51)	145 (13.51)	
**Marital status**				
Married/couple	826 (76.98)	647 (60.30)	179 (16.68)	(4.98,2), *P* = 0.08
Single	181 (16.87)	147 (13.70)	34 (3.17)	
Divorced/Separated/Widowed	66 (6.15)	59 (5.50)	7 (0.65)	
**Education**				
Illiterate	58 (5.41)	54 (5.03)	4 (0.37)	(17.42,5), *p* < .05
Primary education	144 (13.42)	127 (11.84)	17 (1.58)	
secondary education	155 (14.44)	117 (10.90)	38 (3.54)	
Post-secondary non-tertiary education	379 (35.32)	290 (27.03)	89 (8.29)	
First stage of tertiary education	282 (26.28)	220 (20.50)	62 (5.78)	
Second stage of tertiary education	55 (5.13)	45 (4.19)	10 (0.93)	
**Household income**				
1 (Lowest level)	83 (7.74)	65 (6.06)	18 (1.68)	(0.99, 4), *P* = .91
2	289 (26.93)	230 (21.44)	59 (5.50)	
3	565 (52.66)	446 (41.57)	119 (11.09)	
4	122 (11.37)	101 (9.41)	21 (1.96)	
5 (Highest level)	14 (1.30)	11 (1.03)	3 (0.28)	
**Social class (subjective)**				
Upper	17 (1.58)	13 (1.21)	4 (0.37)	(16.74, 4), *P* < .05
Upper-middle	312 (29.08)	257 (23.95)	55 (5.13)	
lower middle	383 (35.69)	282 (26.28)	101 (9.41)	
Working	206 (19.20)	179 (16.68)	27 (2.52)	
Lower	155 (14.45)	122 (11.37)	33 (3.08)	
**Occupation**				
Housewife	376 (35.04)	329 (30.66)	47 (4.38)	
Self-employed	336 (31.31)	244 (22.47)	92 (8.57)	
Employee	128 (11.93)	85 (7.92)	43 (4.01)	
Retired	87 (8.11)	73 (6.80)	14 (1.30)	
Worker	59 (5.50)	42 (3.91)	17 (1.58)	(49.98, 7), *P* < .001
Student	45 (4.19)	42 (3.91)	3 (0.28)	
Unemployed	28 (2.61)	25 (2.33)	3 (0.28)	
Others	14 (1.30)	13 (1.21)	1 (0.09)	
**Perceived social support**				
Family	20 (19,21)	20 (19,21)	20 (18,21)	3.34, *P* < .001
Friends	18 (16,20)	18 (16,20)	19 (17,20)	−2.77, *P* < .01
Significant Other person	20 (19,21)	20 (19,21)	20 (18,21)	2.60, *P* < .01

*P* values are based on χ2 test for the categorical variables and Mann-Whitney U test for the continuous variables, *df* Degree of freedom

Age of the participants ranged from 18 to 89 years (median = 38, interquartile range = 30–51). Four of five participants reported that they completely (309,28.80%) or mostly (544,50.70%) isolated themselves from people outside their households, while, 184 (17.15%) described themselves as somewhat isolated, 24 (2.24%) as a little isolated and 12 (1.12%) not isolated at all. Overall, 220 (20.50%) of the participants reported poor compliance with stay at home orders. Significant differences were seen in sociodemographic characteristics between the participants with good and those with poor compliance. However, no significant differences were observed in marital status and levels of income between the two groups.

Table 2 summarizes results of multivariate logistic regression analysis as odds ratios and 95% confidence intervals.

**Table 2:** Logistic regression models predicting odds of being non-compliant with self –isolation

Independent variable	Model 1	Model 2
Age (centered at mean)	.985 (.973–998)**	.988 (.976–1.001)*
**Sex**		
Female (ref)		
Male	2.457 (1.792–.3.368)***	2.464 (1.789–3.394)***
**Marital status**		
Single (unmarried, divorced, widow) (ref)		
Married/couple	1.715 (1.139–2.580)**	1.782 (1.176–2.698)***
**Education**		
Low	.739 (.492–1.112)	.727 (.477–1.108)
Medium (ref)		
High	.959 (.664–1.385)	1.002 (.687–1.461)
**Household income**		
1 (Lowest level)	1.214 (.676–2.182)	1.277 (.701–2.326)
2	.916 (.634–1.324)	.964 (.662–1.403)
3 (ref)		
4	.822 (.478–1.414)	.908 (.524–1.572)
5 (highest level)	.989 (.261–3.747)	.897 (.234–3.433)
**Social Class**		
Low	1.371 (.953–1.971)*	1.370 (.948–1.979)*
High (ref)		
**Perceived Social support**		
Family		.874 (.803–.950) ***
Friends		1.147 (1.076–1.222)****
Significant other person		.926 (.849–1.010) *

Odds ratio with 95% confidence interval are displayed

*P < .1 **P < .05 *** *p* < .005 **** *P* < .001

Model 1 indicates that the odds of reporting poor compliance with stay at home orders were significantly higher for men (*OR = 2.457, 95% CI = 1.792, 3.368, p < .001*) and married respondents (*OR = 1.715, 95% CI = 1.139, 2.580, p < .05*). No significant associations were found for the socioeconomic factors, however, there was a trend for participants with lower subjective social classes to be more likely to report poor compliance (OR *= 1.371, 95% CI = .953, 1.971, p = .089).*

**Fig. 1:**
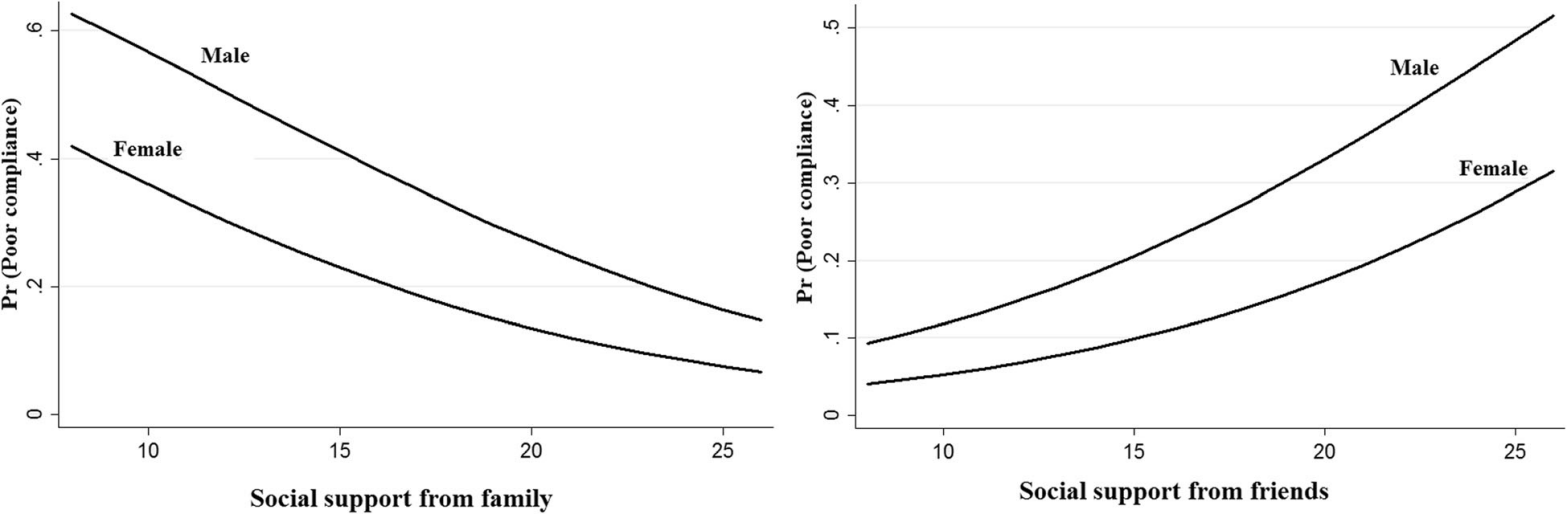
Marginal plots of the effect of perceived social support from family and friends on the poor compliance with stay at home orders

Model 2 suggested that when controlling for demographic and socioeconomic factors, perceived social support from the family was associated with an 12.6% lower odds of reporting poor compliance with self-isolation (*OR = .874, 95% CI = .803, .950, p < .005)*. Interestingly, perceived social support from friends was associated with a 14.7% higher odds of reporting poor compliance (*OR = 1.147, 95% CI = 1.076, 1.222, p < .001*). Participants, who perceived more support from a significant other, were less likely to report poor compliance with self-isolation; however, the result was not statistically significant at 0.05 level (*OR = .926, 95% CI = .849, 1.010, p = .084*). Figure 1 shows the marginal relationship between perceived social support from family and friends and the odds of reporting poor compliance with self-isolation.

## Discussion

The aim of this study was to investigate factors affecting people’s compliance with stay-at-home advice during the current COVID-19 pandemic in Iran. The pandemic occurred during Nowruz (the Persian New Year) holidays, starting from March 19, 2020, and extending for 2 weeks. Nowruz is traditionally time to leave homes for shopping, traveling, and visiting relatives. However, COVID-19 has transformed all the traditions this year [2, 4].

The lack of vaccines or effective treatments for COVID-19 have significantly challenged control of the disease spread. Recent evidence suggests that these types of diseases can include serious social, psychological, and economic consequences. Mashhad, with a population of 3،012،090 individuals, is the second largest holy city in the world, attracting more than 20 million pilgrims and tourists annually especially during Nowruz [27]. In large metropolises such as Mashhad, the importance of limiting outbreaks before their widespread transmissions is a high priority for public health policy makers and planners. Results have shown that most of the people have adopted self-isolation during recent Nowruz in Mashhad. However, nearly one-fifth of the participants had poor compliance with stay at home orders.

Clear social gradients were not found in people’s compliance with stay-at-home directives. However, those with lower subjective social class showed higher odds of non-compliance to social-isolation. People have been asked to practice social distancing as well as economic distancing. Due to numerous economic problems in Iran, general quarantine and strict social distancing include economic hardship for poor people such as those relying on informal labors with no possibilities of social distancing practices [2, 4].

In the present study, the major explanatory factor included perceived social support. The literature suggest positive effects of supportive relationships with other people on promotion of healthy behaviors, as health promotion programs often use social support to change or maintain certain behaviors [6, 10, 17, 18, 28].

Interestingly, social support was found to be both a fostering and hindering factor dependent on the source of support. Participants who perceived more support from their family members were more likely to comply with stay-at-home advices. In contrast, those who perceived more support from friends were more likely to be noncompliant. It appears that close family members may have helped to reinforce the social distancing directive and promoted adherence. Conversely, individuals who rely strongly on the support of friends may have felt greater pressure to leave their homes to socialize, a pressure that may have been amplified as a social norm by some friendship groups.

Studies have shown that different sources of support may have differential effects on health behaviors and outcomes. Researchers have reported that social support from family members is strongly associated with health-related behaviors. However, in some instances, social support could have negative consequences. For example, friends and family, through normative influences, may promote unhealthy behaviors and discourage healthy lifestyles [28–30].

A large and growing body of research has indicated that the family, as a supportive network, plays a significant role in shaping health behaviors [31]. Family is one of the key factors that shapes and affects personal health attitudes, beliefs and behaviors. Family members may model positive health care behaviors or serve as sources of support in crises such as quitting alcohol and caffeine during pregnancy, quitting smoking, and adopting preventive measures [32].

The mechanisms; through which, various aspects of the family relationships (e.g., parental statuses, affectional closeness and obligations) affect health behaviors, have been described via social control theory [31, 32]. Social control theory hypothesizes that family relationships affect health behaviors through indirect and direct control mechanisms. Indirect social control acts through the self-enforcement of norms. Individuals with positive family ties feel a greater sense of responsibility for themselves. Furthermore, families who motivate individuals to practice improve their health behaviors [33]. Support from and accountability to family may directly facilitate changes in behaviors through physical interventions (e.g., preparing special meals), supportive behaviors (e.g., supporting exercise adoptions and routine contacts between the family members who are physically separated) and social sanctions (e.g., threatening to end a marriage if excessive alcohol consumption continues [32].

Although the majority of the literature consistently suggests the positive influence of social support from family on health behavior, the literature on the link with friend and peer support is mixed. A number of researchers have reported that social support provided by network members may also have potential adverse effects on health behaviors [34–38]. Relationships with risk-taking friends and peers can lead to negative health behaviors like alcohol and drug use [30, 31, 39], risky sexual behaviors [37], unhealthy eating behaviors [40], and suicidal behavior [36]. This “social contagion” of negative health-related choices and behaviors may be explained partly by social norms theory. Accordingly, unintended negative consequences of social support from friends and peers may be due to group conformity, where individuals feel pressured to adapt their behavioral norms to match those of their social network [30, 38, 41].

Furthermore, previous research suggests that the effects of social support from friends may be different by the nature of the crisis and the timing of the social support [35], thus further in-depth research is needed to explain the mechanisms by which social support from friends in COVID-19 context can hinder compliance with stay at home directives. The possibility of reverse causation also requires further study.

A limitation in this study was the use of a single-question to identify levels of compliance with stay-at-home directives during the COVID-19 outbreak. This self-report measure was used due to the lack of validated measures of voluntary social isolation [26]. However, as mentioned in the method section, participants were also asked how many times they leaved home for different purposes during the last week. A spearman’s correlation was run to assess the relationship between the stay-at-home compliance (the five-point single-item scale) and frequency of leaving home for all purposes. There was a moderate negative correlation between two variables, which was statistically significant, r_s_ = − .54, *p* < 0.0001.

Another limitation was that the use of a landline phone survey might have increased the possibility of selection bias and overrepresentation of participants with high socioeconomic status because houses with multiple landlines were more likely to be selected and those without landlines (including nearly 3% of the houses according to 2016 reports by Iran Census) were excluded.

Despite these limitations, this study provides valuable insights into some key factors influencing compliance with social distancing orders during the COVID-19 pandemic, for families, policymakers and health service managers. Specifically, results highlighted that compliance with self-isolation may be affected differently by different aspects of people’s social networks.

## Conclusions

The results of this study suggest that public health messaging may need to emphasize the role that friends and families can play in helping to protect those in their friendship/family groups by promoting compliance with social distancing. Further in-depth studies are recommended to evaluate how this kind of messaging can most effectively encourage people to engage in social distancing practices. In addition, it would be valuable to assess social support and compliance with social distancing orders in other countries to evaluate whether the association reported here are found in other countries and cultures.

## Data Availability

The dataset for this study is available from the corresponding author on reasonable request.

## Abbreviations

ACECR: Academic center for education, culture and research
ISCED: International standard classification of education
ISPA: Iranian students polling agency
MSPSS: Multidimensional perceived social support scale
WHO-EMRO: WHO regional office for the eastern mediterranean
